# Efficacy of decellularized meniscus xenogeneic substitutes from sheep and camels compared to human menisci

**DOI:** 10.1002/btm2.70161

**Published:** 2026-07-28

**Authors:** Mohammad Reza Khakzad, Saeed Vafaei‐Nezhad, Tahereh Talaei‐Khozani, Mohammad Mehdi Hassanzadeh‐Taheri, Mohsen Rezaeipour, Hamid Hashemi, Mehri Shadi, Mohammad Afshar

**Affiliations:** ^1^ Innovative Medical Research Center and Department of Immunology Faculty of Medicine, Mashhad Medical Science, Islamic Azad University Mashhad Iran; ^2^ Department of Anatomy Faculty of Medicine, Birjand University of Medical Sciences Birjand Iran; ^3^ Cellular & Molecular Research Center, Birjand University of Medical Sciences Birjand Iran; ^4^ Histomorphometry and Stereology Research Center, Anatomy Department Shiraz University of Medical Sciences Shiraz Iran; ^5^ Tissue Engineering Lab, Department of Anatomical Sciences School of Medicine, Shiraz University of Medical Sciences Shiraz Iran; ^6^ Department of Orthopedic Surgery Imam Reza Hospital, Birjand University of Medical Sciences Birjand Iran; ^7^ Department of Mechanical Engineering Birjand University of Technology Birjand Iran; ^8^ Medical Toxicology Research Center, Department of Pharmacology, Mashhad University of Medical Sciences Mashhad Iran

**Keywords:** biocompatibility, decellularization, meniscus, regeneration, tissue engineering

## Abstract

Meniscal damage treatment is challenging due to limited healing potential. Decellularized scaffolds offer tissue engineering potential, but their efficacy depends on maintaining extracellular matrix structure, host integration, biocompatibility, and minimizing immune response. This study aims to compare decellularized menisci from humans, sheep, and camels for regenerative applications. Three decellularized menisci underwent rigorous decellularization, assessed through DNA and glycosaminoglycan quantification, histological examination, scanning electron microscopy, mechanical moduli, degradation kinetics, biocompatibility, cytotoxicity, fibroblast proliferation potential, immunogenicity assays, and Raman confocal spectroscopy for scaffold constitutions. Histological and DNA analyses confirmed the removal of cellular components, preserving collagen and reducing glycosaminoglycans. The scaffolds were nontoxic. While the ultimate tensile strength remained unchanged, the compressive modulus decreased significantly after decellularization. The camel meniscus showed compressive properties comparable to those of the intact human meniscus. Decellularized menisci in all three species had greater porosity and collagen fiber alignment, and the collagen content of decellularized camel meniscus was comparable to that of native human meniscus. In a subcutaneous xenogeneic rat model, decellularized camel meniscus scaffolds exhibited a favorable host response compared to human and sheep scaffolds, characterized by reduced inflammatory infiltration, robust fibroblast migration into the scaffold interior, and slower degradation after 4 weeks. Decellularized camel meniscus represents a preclinical xenogeneic scaffold candidate for meniscus regeneration, particularly in regions where porcine tissues are inaccessible and human allografts are scarce. Its favorable preclinical remodeling profile and dimensional compatibility with human tissue support further validation in large‐animal intra‐articular models prior to clinical translation.

## Graphical abstract

Comparative analysis of decellularized meniscal scaffolds derived from camel, human, and sheep tissues was performed to evaluate extracellular matrix preservation, mechanical integrity, and biocompatibility, supporting their potential for meniscal repair and regeneration.

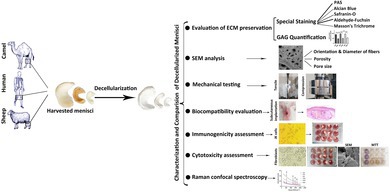

**Translational Impact Statement**

This study identifies decellularized camel meniscus as a regionally feasible xenogeneic scaffold for meniscus regeneration in the Middle East, where porcine sources are inaccessible and human allografts are limited. In preclinical screening, CDM demonstrated favorable host remodeling characteristics, including reduced inflammation and enhanced fibroblast integration supporting its potential as a pragmatic alternative to synthetic prostheses following total meniscectomy.

## 1 INTRODUCTION

### 1.1 Meniscus biology and current treatment strategies

One of the most common intra‐articular knee injuries is meniscal tears. They usually occur in both older people and those who are more physically active. Fibrocartilaginous structures are critical for load bearing, joint stability, and shock absorption in the knee joint.^1^ The capacity of the meniscus to self‐heal is limited, especially after injury, due to its avascular inner zone, which increases the risk for worsening joint disorders such as osteoarthritis.^2^ Current clinical practices, such as partial meniscectomy, meniscal repair, and meniscus allograft transplantation, are often associated with variable success rates, limited donor tissue, and long‐term consequences for biomechanics and joint degeneration.^3^

### 1.2 Decellularized extracellular matrix scaffolds for meniscus regeneration

Through the development of biological substitutes with the purpose of restoring both the structural and functional aspects of the native meniscus, tissue engineering has emerged as an exciting approach for addressing these limitations.^4^ In particular, decellularized extracellular matrix (ECM) plays a significant role in the engineered meniscus due to its ability to preserve native structural and biochemical signals while eliminating the cells and cell debris that trigger immune responses. These scaffolds offer a novel approach for enhancing host cell migration, tissue regeneration, and integration within the biological frameworks of engineered systems.^5^ The decellularization protocols usually include a combination of chemical removal, enzymatic digestion, and mechanical elimination of the cells without disrupting the architecture of the ECM.^6^ Our previous work introduced an optimized protocol for rabbit meniscus decellularization, since all significant biomechanical and histological characteristics of the whole decellularized meniscus, including the collagen fiber orientation, glycosaminoglycan (GAG) content, and mechanical function, are preserved. Furthermore, the viability of seeded chondrocytes remains high when cultured on these decellularized scaffolds.^7^ However, successful clinical application of such xenogeneic or allogeneic scaffolds requires a detailed understanding of interspecies variation in ECM composition, biocompatibility, and mechanical strength.^8^, ^9^

### 1.3 Species selection for xenogeneic meniscal scaffolds

Examining the various features and functions of different species clarifies the need to identify the animal equivalent with the potential to functionally replace human menisci and accelerates the advancement of strategies for preclinical tissue engineering models. Anatomical studies of menisci in sheep, pigs, dogs, and humans have shown that sheep menisci are structurally and mechanically similar to those of humans; thus, they can be accurately used for surgical and biomechanical assessments. Porcine menisci also exhibit favorable cellular and compositional similarities to human menisci, though their larger dimensions limit direct anatomical equivalence.^10^ The structural compositions of types I and II collagen and GAG contents in sheep, pigs, goats, and dogs are comparable to those in humans. Pigs have similar distributions of collagen and GAG, and the histological morphology of fibrocartilage aligns with that of humans. The vascular patterns in the meniscus are similar, with nearly equivalent infiltration in sheep and pigs. The distribution patterns of innervation and the types of mechanoreceptors are similar to those of the human meniscus.^11^ Among animal models tested, the sheep meniscus demonstrates the closest biomechanical match to human menisci in terms of stiffness, residual force, and relative compressibility. Histologically, the Safranin O staining patterns in sheep menisci are most similar to those in human tissues.^12^ Ovine and humans have similarities in anatomy, composition, and ECM organization. The compressive and tensile mechanical properties, including the stiffness and ultimate tensile load values, are comparable.^13^ Humans and equine menisci have common meniscal pathologies. Menisci are composed of the same network of type I collagen, meniscal cells, and ECM components.^14^ Goats and pigs could be suitable models for replacing the human meniscus because of their anatomical structures. The pig meniscus is usually recommended for both in vitro and in vivo studies because of the position and mechanical horn attachment.^15^ Furthermore, the decellularized medial porcine meniscus has shown compressive moduli and permeability values within the range of human meniscal tissue, thus making it a suitable biological scaffold for partial meniscal replacement.^16^ Minipigs closely resemble human menisci in terms of shape, size, and elasticity. They have the same collagen network and GAG content, along with scattered cells. The circumferential and radial tensile properties are comparable, which supports their use for translational meniscus research.^8^ Large animal models offer the greatest translational value for use in human medical research. Yet, systematic cross‐species comparisons of whole decellularized meniscal scaffolds, benchmarked against human tissue, remain limited. Few studies have systematically compared the decellularization of whole meniscal tissues across species, particularly regarding their structural, biochemical, and immunological properties relative to human scaffolds. Given regional constraints in Iran and the Middle East, where porcine tissues are inaccessible due to socio‐cultural restrictions, we selected camel and sheep menisci as regionally available xenogeneic alternatives. Camel menisci were chosen for their closer dimensional correspondence to human tissue and favorable biomechanics compared to oversized bovine alternatives, while sheep menisci serve as a well‐characterized comparative model with established structural and mechanical parallels to humans. To address this translational gap and evaluate regionally feasible scaffold candidates, we conducted a comprehensive comparative evaluation of decellularized menisci from human, camel, and sheep sources. Through integrated analyses, including histology, DNA/GAG quantification, SEM, mechanical testing, and in vitro/in vivo biocompatibility assessments, this study aims to elucidate interspecies differences and identify optimal candidates for meniscal reconstruction.

## 2 MATERIALS AND METHODS

### 2.1 Animal model

For the in vivo biocompatibility assessment, 18 male Sprague‐Dawley rats (250–300 g) were obtained from the animal facility of Birjand University of Medical Sciences. All procedures were conducted in accordance with the ARRIVE 2.0 guidelines and the “Guideline for the Care and Use of Laboratory Animals in Iran,” under the supervision of the Research Centre of Experimental Medicine. The study protocol was approved by the Ethics Committee of Birjand University of Medical Sciences (approval code: IR.BUMS.REC.1400.085). Animals were housed at constant room temperature (22 ± 2°C) with a 12‐h light/dark cycle and ad libitum access to food and water. Rats were randomly assigned to three scaffold groups (camel decellularized meniscus (CDM), human decellularized meniscus (HDM), sheep decellularized meniscus (SDM); *n* = 6 per group), with three animals per group harvested at each of two timepoints (1 and 4 weeks) for histological analysis.

### 2.2 Tissue collection and decellularization

Fresh medial and lateral menisci were harvested from camels and sheep post‐slaughter from a local abattoir, whereas human menisci were obtained from patients undergoing total knee arthroplasty. The anatomical dimensions of each meniscus type are summarized in Table 1. All specimens were measured using a digital Vernier caliper (Louisware, Dubai) prior to processing. The meniscal tissues were rinsed with phosphate‐buffered saline (PBS) and stored at −20°C until further processing.

**TABLE 1:** Dimensions of camel, human, and sheep meniscus.

	Camel	Human	Sheep
Outer circumference (mm)	105–115	80–90	45–50
Central width (mm)	20	10	7
Peripheral height (mm)	10	5.5–6	5

The entire menisci were immersed in 10 mM Tris–HCl solution for 24 h, followed by four freeze–thaw cycles over 48 h to disrupt cellular membranes. Subsequently, the tissues were incubated in 0.25% (w/v) trypsin (Sigma‐Aldrich) in PBS for 24 h, followed by 1% (v/v) Triton X‐100 (Sigma‐Aldrich) in distilled water for 24 h. Samples were then treated with 1.5% (w/v) sodium lauryl ether sulfate (SLES; Kimia Sanaat Ataman Co., Iran) and 0.3% (w/v) ethylenediaminetetraacetic acid (EDTA; Parstous, Iran) in PBS for 72 h under continuous stirring. A second 24‐h incubation with 1% (v/v) Triton X‐100 in distilled water was performed, followed by a final immersion in 0.05% (w/v) trypsin–EDTA in PBS for 48 h. To increase scaffold porosity and remove residual cellular material, the tissues were incubated in 2% (v/v) Triton X‐100 and 1.5% (v/v) peracetic acid in distilled water for 72 h. Continuous stirring was maintained throughout all decellularization steps. Following decellularization, scaffolds were allocated based on downstream applications: freeze‐dried scaffolds were used for mechanical testing, SEM analysis, degradation/hydration assays, and Raman spectroscopy, while fresh (non‐lyophilized) scaffolds were reserved for histological evaluation and in vitro/in vivo biocompatibility assessments. Freeze‐drying was performed using a laboratory‐scale lyophilizer (Model PFD‐5012; condenser capacity: 12 L, −50°C). Samples were pre‐frozen at −80°C for 12 h (controlled cooling rate: 1°C/min), followed by primary drying at 0.1 mbar and −40°C for 24 h, and secondary drying at 0.05 mbar and 25°C for 12 h.

### 2.3 Characterization of the decellularized meniscus

#### 2.3.1 Verification of cellular removal and DNA quantification

The decellularized meniscal scaffolds were fixed in 4% (w/v) paraformaldehyde in PBS and embedded in paraffin for histological analysis. Serial sections (5 μm thickness) were stained with hematoxylin and eosin (H&E) to confirm the removal of cellular components. For DNA quantification, 25 mg of each freeze‐dried scaffold (*n* = 3 per group) was processed using a tissue genomic DNA extraction kit (FavorPrep, Taiwan). DNA concentration was evaluated by spectrophotometry (NanoDrop Technologies, Wilmington, DE, USA) at an absorbance of 260 nm. Successful decellularization was defined as residual DNA content <50 ng/mg dry weight, consistent with international criteria for decellularized tissues.^17^

#### 2.3.2 Histological evaluation of ECM preservation

The retention of ECM components was assessed using specific histological stains, including Safranin‐O, Masson's Trichrome, Periodic Acid‐Schiff (PAS), Alcian Blue, and Gomori's aldehyde‐Fuchsin, to evaluate GAGs, polysaccharides, collagen fibers, and elastic fibers in both native and decellularized meniscal tissues.

#### 2.3.3 GAG quantification and collagen fiber analysis

The GAG content of lyophilized meniscal tissues was quantified using the dimethylmethylene blue (DMMB) assay. The DMMB reagent was prepared by dissolving 16 mg of DMMB, 3.04 g of glycine, and 1.6 g of NaCl in distilled water, adjusting to pH 3.0 with 0.1 M acetic acid, and bringing the final volume to 100 mL. Each lyophilized tissue sample (100 mg, *n* = 3 per group) was minced and digested enzymatically overnight at 56°C in a solution containing 0.5 mg/mL (0.05% w/v) proteinase K in Tris–HCl buffer (pH 8.0). GAG content was determined by mixing 20 μL of the digested sample was mixed with 200 μL of DMMB dye in a 48‐well microplate, and the absorbance was measured immediately at 656 nm using a microplate reader. Collagen fiber staining intensity was quantified from Masson's Trichrome‐stained images using ImageJ software,^18^ analyzing three non‐overlapping fields per sample at 100× magnification.

#### 2.3.4 Scanning electron microscopy analysis

The microstructural features of the decellularized and intact menisci (*n* = 3 per group), including the orientation and diameter of the collagen fibers, as well as the pore size and porosity, were investigated by scanning electron microscopy (SEM, TESCAN, Brno, Czech Republic). The samples were fixed with 2.5% (v/v) glutaraldehyde in 0.1 M phosphate buffer (pH 7.4), followed by lyophilization. The SEM images were evaluated using ImageJ software.

#### 2.3.5 Mechanical testing

The ultimate tensile strength (UTS) and compression moduli of both the hydrated decellularized and native meniscal samples (*n* = 3 per group) were measured using a universal testing machine (STM‐50; Santam, Iran). For UTS testing, specimens were oriented parallel to the circumferential collagen fiber direction, the primary load‐bearing axis of native menisci. The average cross‐sectional areas of the camel, human, and sheep menisci were 100, 35, and 20 mm^2^, respectively. Tensile and compression testing was carried out at a strain rate of 3.5 mm/min until failure.^7^ Tangent compressive modulus was calculated as the slope of the stress–strain curve at 10% strain during the loading phase.^19^

#### 2.3.6 Evaluation of biocompatibility and in vitro immunogenicity

For subcutaneous implantation, rats were anesthetized with ketamine (80 mg/kg) and xylazine (5 mg/kg) via intraperitoneal injection. A 1‐cm dorsal incision was made, and one scaffold (5 × 5 mm) was implanted into a dorsal subcutaneous pocket in each animal. Incisions were closed with 4–0 silk sutures, and postoperative analgesia was provided with piroxicam (1 mg/kg, SC) for 48 h. Animals were euthanized by CO_2_ asphyxiation at 1 and 4 weeks (*n* = 3 per timepoint), and explants were fixed in 4% paraformaldehyde for H&E histology. Histomorphometric analysis quantified inflammatory cells (peripheral region) and fibroblasts (central scaffold) across five non‐overlapping fields per sample at 100× magnification; remaining scaffold area was measured using ImageJ software.

#### 2.3.7 Degradation rate evaluation

In vitro enzymatic degradation of the scaffolds was assessed using 1% (w/v) trypsin in PBS solution. Briefly, scaffolds (initial weight: W_0_; *n* = 3 per group) were incubated in trypsin at 37°C. At predetermined time points (daily during week 1, then weekly up to day 28), samples were removed, dried, and weighed. The remaining mass (%) was calculated as follows:

$$\text{Remaining mass}\;\left(\%\right)\;:\left(W_{t}/W_{0}\right)\times 100$$

where *W*_0_ represents the initial scaffold weight and *W*_*t*_ represents the remaining scaffold weight at each time point.

#### 2.3.8 Swelling ratio evaluation

To evaluate scaffold hydration behavior under physiologically relevant conditions, dry scaffolds (*n* = 3 per group) were initially weighed (Wd) and immersed in PBS (pH 7.4) at 37°C. The scaffolds were weighed at predetermined time intervals over 120 min following gentle removal of excess surface water. The swelling ratio (%) was calculated as follows:

$$\text{Swelling ration}\;\left(\%\right)\;:\left[\left(W_{t}-W_{d}\right)/W_{d}\right]\times 100$$

where *W*_*d*_ is the initial dry weight and *W*_*t*_ is the scaffold weight at each time point.

#### 2.3.9 Cytotoxicity assessment

For in vitro biocompatibility assessment, decellularized scaffolds were UV‐sterilized, pre‐hydrated in complete medium for 24 h at 37°C, and seeded with NIH 3T3 fibroblasts (1 × 10^5^ per scaffold; *n* = 3/group) in 1 mL DMEM high‐glucose supplemented with 10% (v/v) fetal bovine serum (FBS), 1% (v/v) penicillin–streptomycin, and 2 mM L‐glutamine. Cell viability was evaluated after 24 and 48 h via MTT assay: scaffolds were incubated with 1 mg/mL MTT (M5655, Sigma‐Aldrich) in phenol red‐free medium for 3 h at 37°C/5% CO_2_, formazan crystals were solubilized with DMSO, and absorbance was measured at 590 nm. All measurements were performed in triplicate and normalized to cell‐free blanks.

#### 2.3.10 In vitro immunogenicity assessment

For in vitro immunogenicity assessment, decellularized meniscal scaffolds (prepared as described above) were co‐cultured with 1 × 10^5^ Jurkat T‐lymphocytes (Clone E6‐1; ATCC® TIB‐152™) suspended in 1 mL of RPMI‐1640 medium supplemented with 10% (v/v) FBS, 1% (v/v) penicillin–streptomycin, and 2 mM L‐glutamine. Cultures were maintained at 37°C in a humidified 5% CO_2_ atmosphere. Jurkat cell proliferation was quantified at 24, 48, and 72 h post‐seeding, and all experiments were performed in triplicate.

#### 2.3.11 Raman confocal microscopy

Raman spectroscopy was employed to analyze the compositional characteristics of both intact and decellularized menisci. Spectra were recorded using a laser with a power of 50 mW at a wavelength of 785 nm. Data were collected over a spectral range of 500–2500 cm^−1^ with a spectral resolution of 4 cm^−1^.

#### 2.3.12 Statistical analyses

Statistical analyses were performed using GraphPad Prism v9.0. Data normality was assessed by the Shapiro–Wilk test and homogeneity of variance by the Brown–Forsythe test. For normally distributed data with equal variances, group comparisons were conducted using one‐way or two‐way ANOVA followed by Tukey's post‐hoc test. Statistical significance was defined as *p*< 0.05 and is indicated directly on figures using asterisk notation (*p< 0.05, **p< 0.01, ***p< 0.001; ns, non‐significant), with complete statistical details provided in figure legends. Data are presented as mean ± standard deviation (SD), with individual data points (*n* = 3/group).

## 3 RESULTS

### 3.1 Decellularization efficiency

**FIGURE 1:**
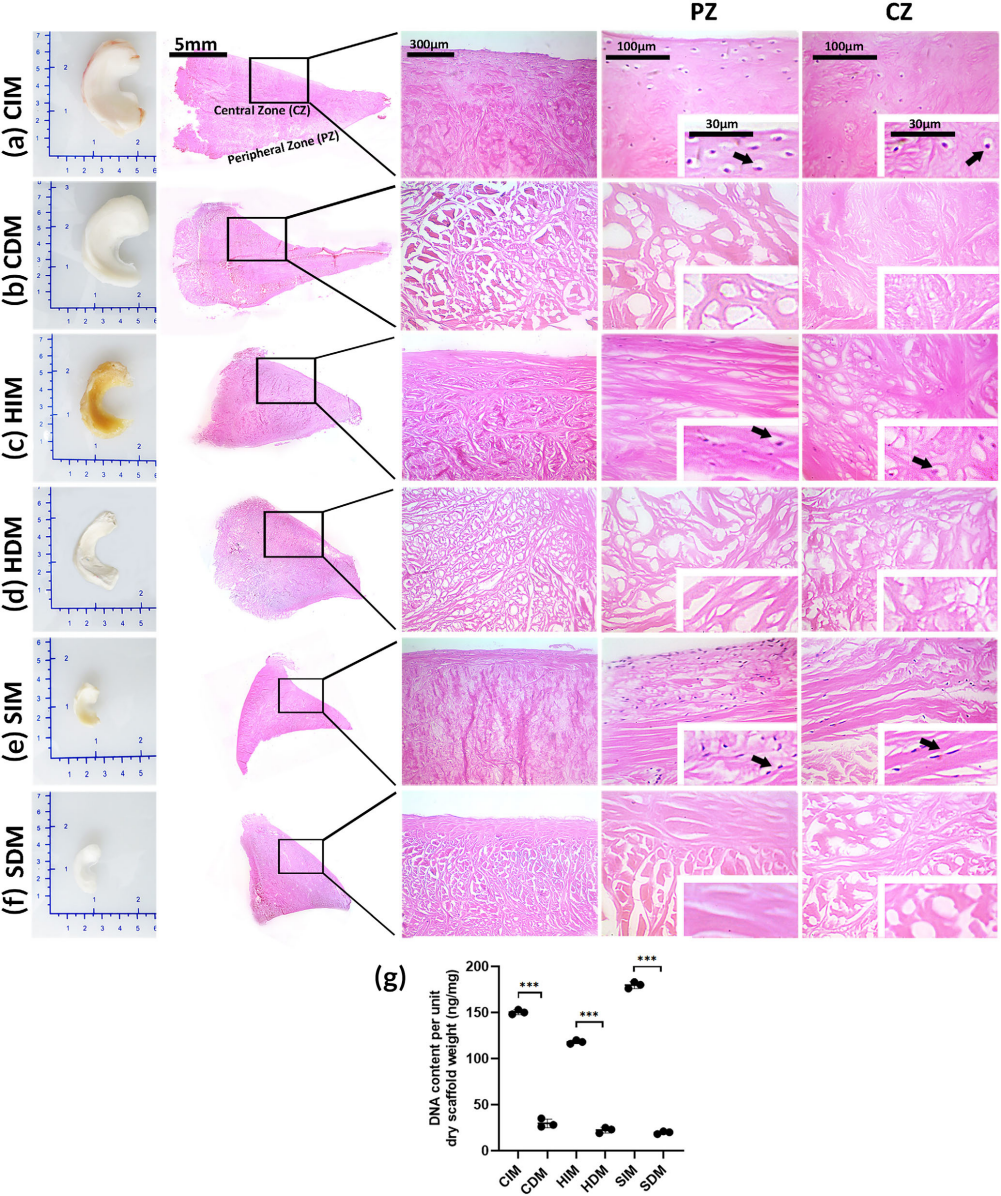
Histological and quantitative assessment of decellularization efficiency in camel, human, and sheep meniscal scaffolds. (a)–(f) Macroscopic views of meniscal samples (first column) and corresponding H&E‐stained histological sections at progressively higher magnifications. The final two columns show the peripheral zone (PZ) and central zone (CZ) of intact and decellularized menisci. Arrows indicate residual cellular nuclei. Magnified views in column 3 represent regions of interest (ROIs) from column 2; exact spatial correspondence is not maintained to optimize visualization of key structural features. (g) DNA quantification. Mean ± SD with individual data points (*n* = 3/group); one‐way ANOVA with Tukey's post‐hoc test (***p< 0.001). CIM, camel intact meniscus; CDM, camel decellularized meniscus; HIM, human intact meniscus; HDM, human decellularized meniscus; SIM, sheep intact meniscus; SDM, sheep decellularized meniscus.

Gross examination of the lyophilized decellularized scaffolds revealed preservation of their natural shape. Histological analysis via H&E staining confirmed successful removal of cell nuclei in both the peripheral and central zones (PZ and CZ). However, some sections presented a minimal number of residual nuclei (Figure 1a–f).

Quantitative DNA analysis further supported the removal of cells from the decellularized scaffolds. The residual DNA content in the scaffolds, measured at 29.67 ± 4.70 ng/mg in CDM, 22.33 ± 3.05 ng/mg in HDM, and 19.67 ± 1.52 ng/mg in SDM, (per dry weight), was significantly lower than that in matched intact menisci (CIM, 150.33 ± 2.51 ng/mg; HIM, 118.00 ± 2.00 ng/mg; and SIM, 179.70 ± 3.51 ng/mg; Figure 1g).

### 3.2 ECM preservation

**FIGURE 2:**
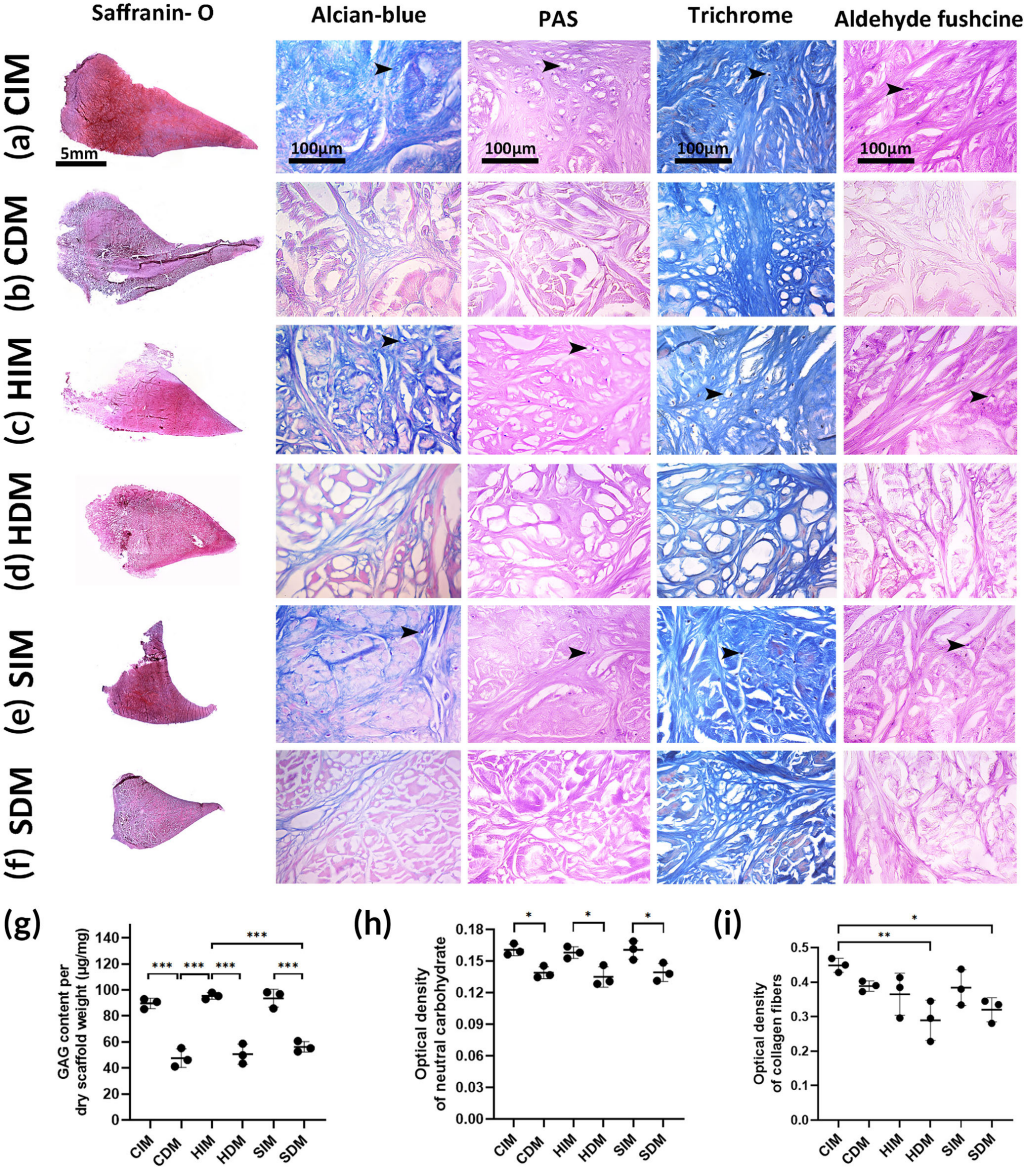
Evaluation of ECM preservation in intact and decellularized menisci via special stains and quantitative analysis. (a)–(f) Safranin‐O, Alcian Blue, PAS, Masson's Trichrome, and Aldehyde‐Fuchsin staining were used to assess GAG, polysaccharide, collagen, and elastic fiber content in intact and decellularized groups. (g) GAG quantification, (h) quantitative analysis of neutral carbohydrate content, and (i) collagen fiber staining intensity across groups. Mean ± SD with individual data points (*n* = 3/group); one‐way ANOVA with Tukey's post‐hoc test (*p< 0.05, **p< 0.01, ***p< 0.001).

Histological staining, including Safranin‐O, Alcian Blue, PAS, Masson's Trichrome, and Aldehyde‐Fuchsin, revealed a decrease in ECM components (Figure 2a–f). Quantitative analysis verified a significant decrease in GAG content in all decellularized groups compared with their corresponding intact tissues (Figure 2g). Both CDM and SDM presented lower GAG contents than HIM did, indicating variable retention of matrix components across the three species. Quantitative assessment of PAS staining verified a significant decrease in neutral carbohydrate content across all decellularized groups compared to intact controls (Figure 2h). Compared with the corresponding intact menisci, the intensity of Masson's Trichrome staining was not significantly lower in the decellularized menisci of the three species, and a marked decrease was detected only in the HDM and SDM groups compared with the CIM group (Figure 2i), likely reflecting inherent interspecies differences in baseline collagen density rather than a processing effect.

### 3.3 SEM morphological and structural analysis

**FIGURE 3:**
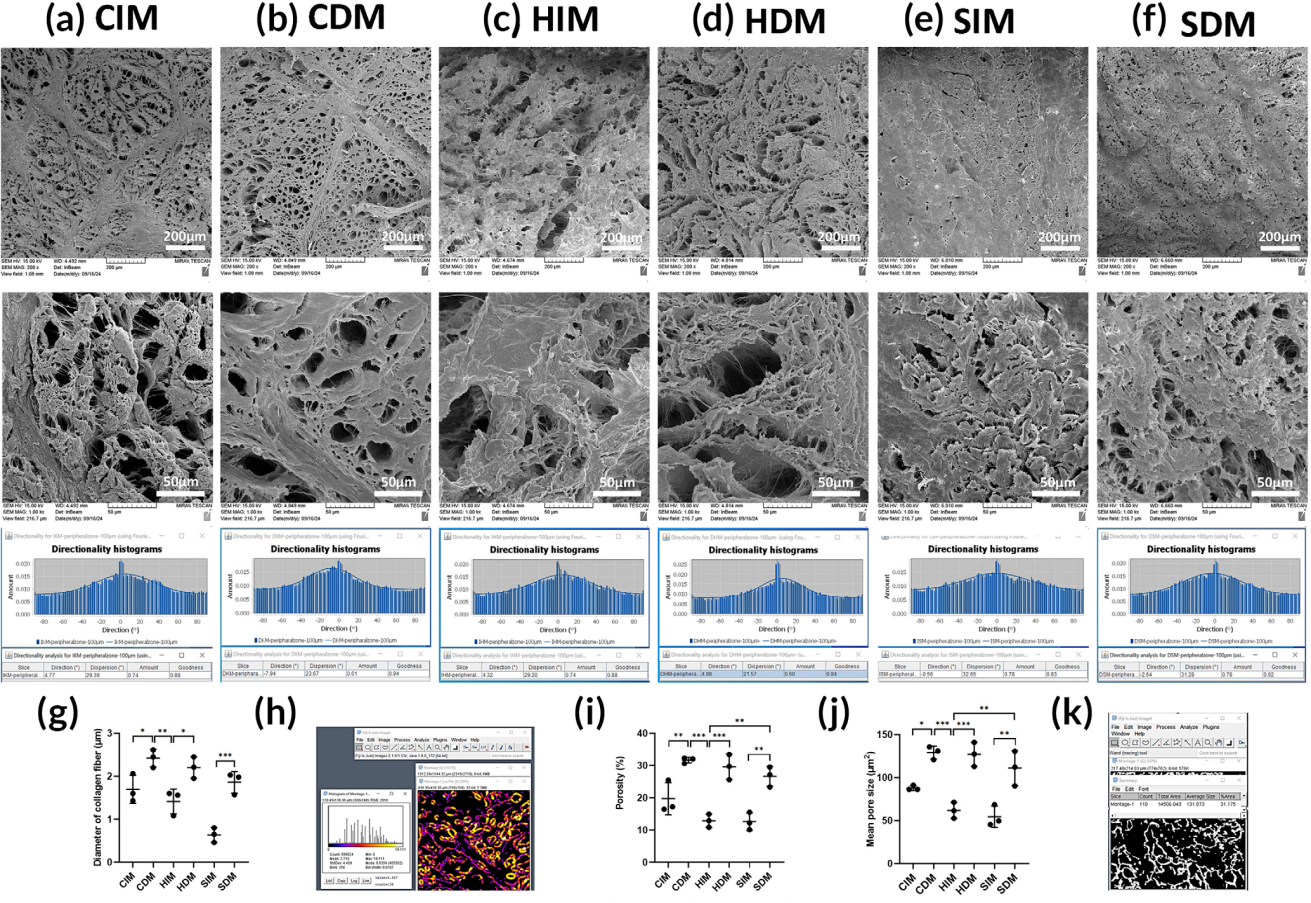
Structural and extracellular matrix characterization of intact and decellularized meniscal scaffolds. (a–f, top and middle rows) SEM images at low and high magnification showing surface morphology and collagen structure in intact (CIM, HIM, SIM) and decellularized (CDM, HDM, SDM) menisci. (a–f, third row) Directionality histograms demonstrating collagen fiber orientation, distribution, and goodness of fit across all groups. (g), (h) Collagen fiber diameter quantification (μm); (i)–(k) porosity (%) and mean pore size (μm^2^) analyzed using ImageJ. Mean ± SD with individual data points (*n* = 3/group); one‐way ANOVA with Tukey's post‐hoc test (*p< 0.05, **p< 0.01, ***p< 0.001).

The collagen fiber orientation and diameter, porosity, and pore size of intact and decellularized meniscal tissues were assessed via ImageJ software to analyze the SEM images (Figure 3a–k). A highly aligned collagen fiber with a goodness‐of‐fit value above 0.8 indicates in directionality histograms across all groups that the anisotropic architecture, including fiber alignment and distribution, was mostly preserved following decellularization (Figure 3a–f, third row). A significant increase in collagen fiber diameter was detected in the CDM (2.42 ± 0.20 μm), HDM (2.20 ± 0.25 μm), and SDM (1.86 ± 0.25 μm) groups compared with their native counterparts CIM (1.69 ± 0.32 μm), HIM (1.41 ± 0.28 μm), and SIM (0.63 ± 0.17 μm). This increased diameter likely results from fibril separation following proteoglycan and GAG removal, permitting compacted fibrils to swell. Notably, the fiber diameters in CDM were significantly greater than those in HIM, although the difference from SDM was not significant (Figure 3g,h).

Compared with the native control scaffolds CIM (19.75% ± 5.00%), HIM (12.92% ± 2.05%), and SIM (12.65% ± 2.72%), decellularized scaffolds presented significant increases in porosity, CDM (31.70% ± 0.89%), HDM (29.63% ± 3.90%), and SDM (26.71% ± 3.10%). These findings confirm the improved permeability and cellular removal of the decellularized scaffolds.

The mean pore size was markedly greater in the decellularized groups CDM (129.10 ± 7.50 μm^2^), HDM (127.10 ± 13.97 μm^2^), and SDM (111.10 ± 20.12 μm^2^) than in the CIM (87.95 ± 2.80 μm^2^), HIM (61.84 ± 9.30 μm^2^), and SIM (54.56 ± 12.30 μm^2^) groups, which may enhance the cell infiltration capacity and nutrient diffusion. Compared with those of HIM, the pore sizes of CDM and SDM were significantly greater (Figure 3i,j).

### 3.4 Mechanical testing

**FIGURE 4:**
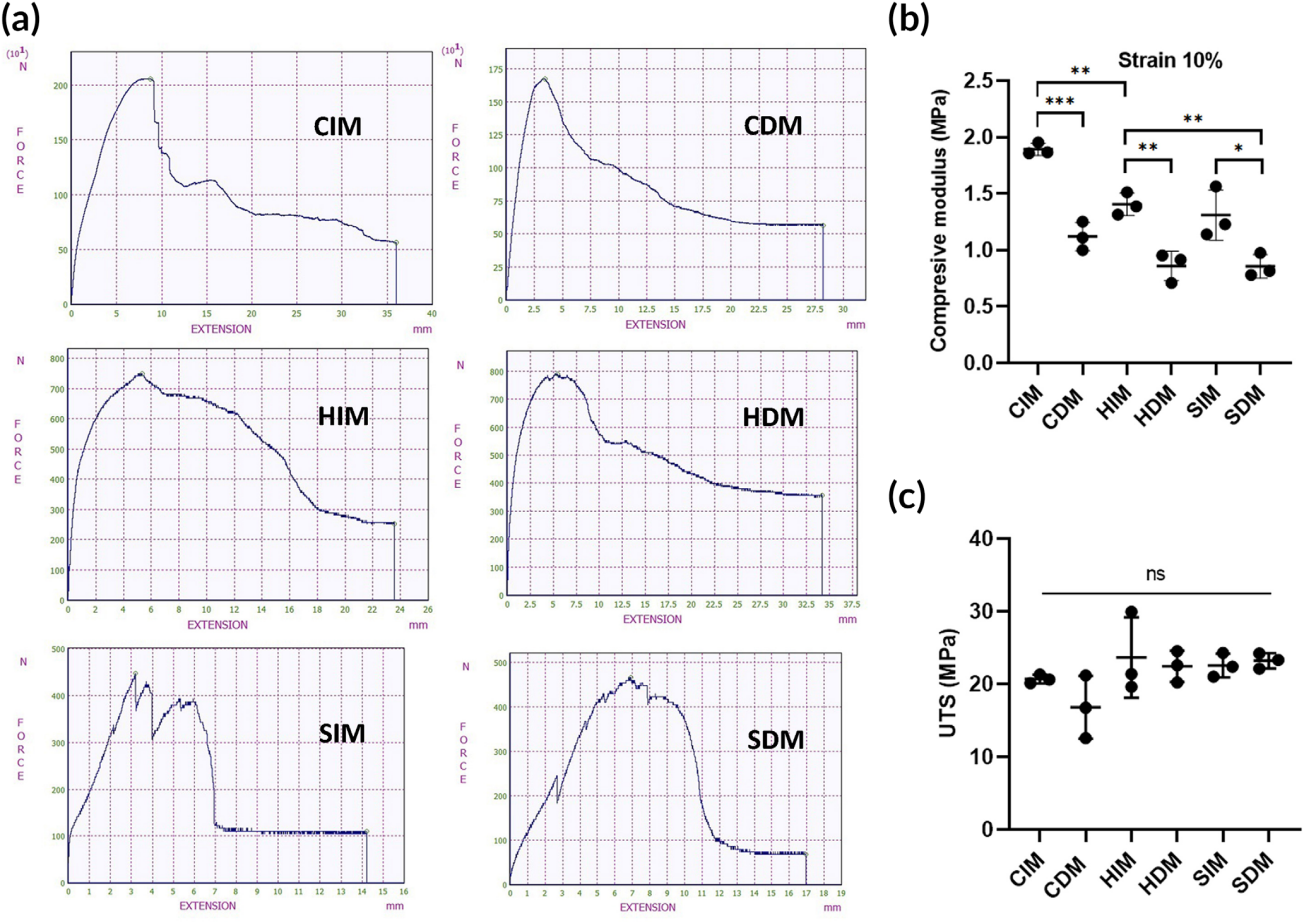
Mechanical characterization of intact and decellularized meniscal scaffolds. (a) Compressive stress–strain curves for hydrated specimens; engineering stress calculated using measured cross‐sectional areas (CIM and CDM: 100 mm^2^, HIM and HDM: 35 mm^2^, SIM and SDM: 20 mm^2^). (b) Tangent compressive modulus at 10% strain. (c) Ultimate tensile strength (UTS) with specimens oriented parallel to circumferential collagen fibers. Mean ± SD with individual data points (*n* = 3/group); one‐way ANOVA with Tukey's post‐hoc test (*p< 0.05, **p< 0.01, ***p< 0.001; ns, non‐significant).

Decellularized menisci presented a notable decrease in the compressive modulus. The measured values were CDM (1.12 ± 0.22 MPa), HDM (0.86 ± 0.13 MPa), and SDM (0.85 ± 0.10 MPa), which were significantly lower than those of the matched intact tissues, CIM (1.89 ± 0.05 MPa), HIM (1.40 ± 0.10 MPa), and SIM (1.30 ± 0.07 MPa). Despite these reductions, no significant difference in compressive modulus was found between CDM and HIM, suggesting retention of clinically relevant compressive properties despite interspecies and processing variations. The UTS was similar in the intact and decellularized samples. This finding suggests that the collagenous architecture was maintained sufficiently to retain tensile integrity following decellularization. Nevertheless, the observed decrease in the compressive modulus of the decellularized scaffolds indicates a partial loss of matrix stiffness post‐decellularization (Figure 4a–c).

### 3.5 In vivo biocompatibility and degradation

**FIGURE 5:**
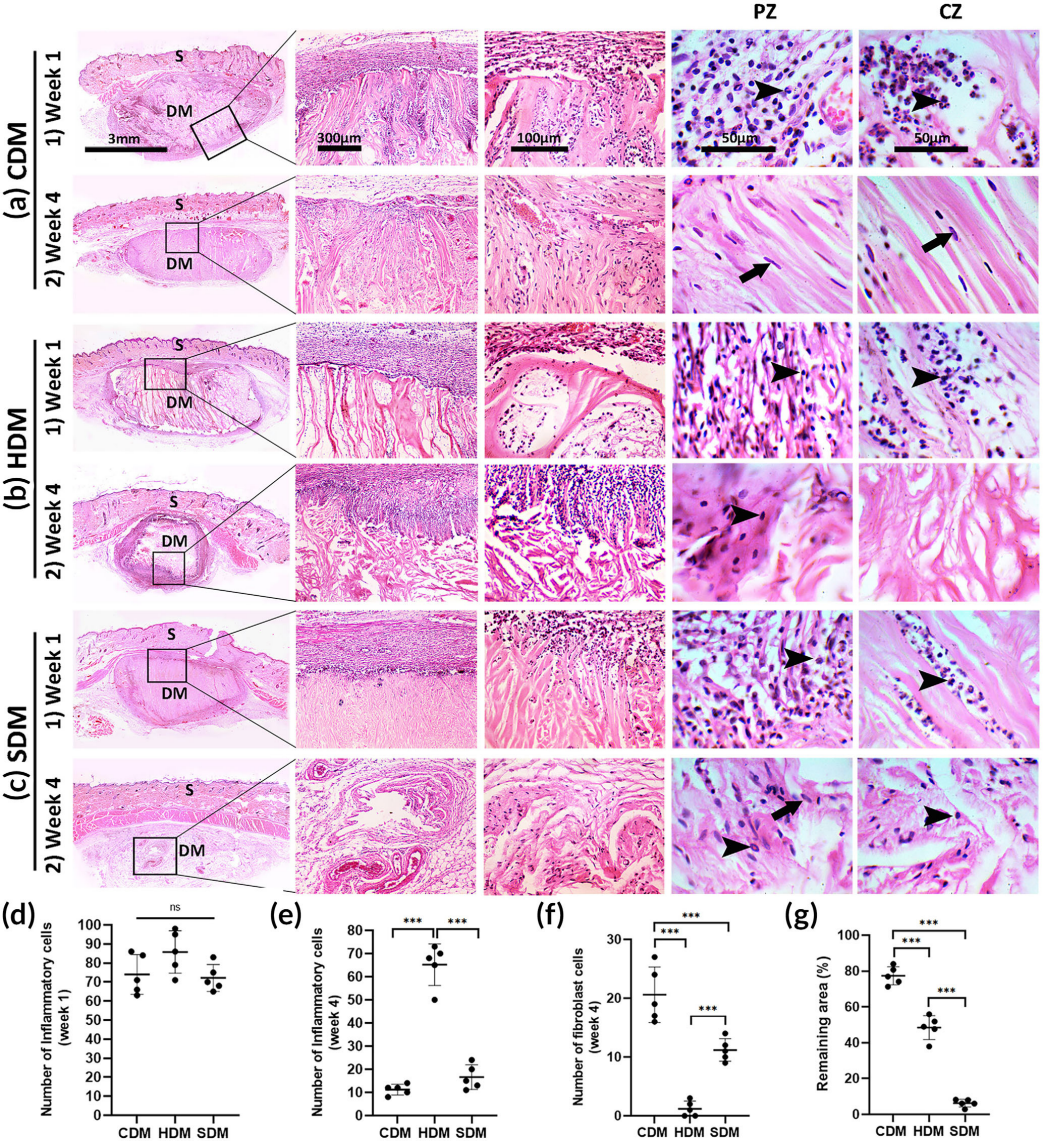
In vivo biocompatibility, cellular infiltration and degradation behavior of decellularized meniscal scaffolds following subcutaneous implantation. (a)–(c) Low‐ and high‐magnification H&E‐stained sections of subcutaneously implanted scaffolds after 1 and 4 weeks showing immune cell infiltration, fibroblast migration, and scaffold degradation (arrows mark fibroblasts; arrowheads indicate inflammatory cells; S, skin; DM, decellularized meniscus). (d) Quantitative analysis of inflammatory cell infiltration in the peripheral region of scaffolds after 1 week; (e) after 4 weeks. (f) Quantitative analysis of fibroblast infiltration within the central scaffold area after 4 weeks. (g) Quantitative analysis of remaining scaffold area (%) after 4 weeks of implantation. Mean ± SD (*n* = 3/group); one‐way ANOVA with Tukey's post‐hoc test (*p< 0.05, **p< 0.01, ***p< 0.001; ns, non‐significant).

At week 1, all scaffolds elicited comparable acute inflammatory infiltration (lymphocytes, neutrophils) in this xenogeneic rat model. By week 4, CDM scaffolds exhibited attenuated inflammation, robust fibroblast migration into the scaffold interior, and progressive remodeling in both peripheral and central zones. In contrast, HDM scaffolds retained persistent periscaffold inflammatory infiltrates without substantial fibroblast infiltration, whereas SDM scaffolds exhibited reduced inflammation (Figure 5a–d). Quantitative histomorphometry confirmed significantly higher residual inflammation in HDM versus CDM/SDM at week 4 (Figure 5e), enhanced fibroblast infiltration specifically in CDM (Figure 5f), and significantly greater remaining scaffold area in CDM (Figure 5g), indicating slower degradation and superior structural stability in this preclinical screening model.

### 3.6 Degradation kinetics and swelling ratio

**FIGURE 6:**
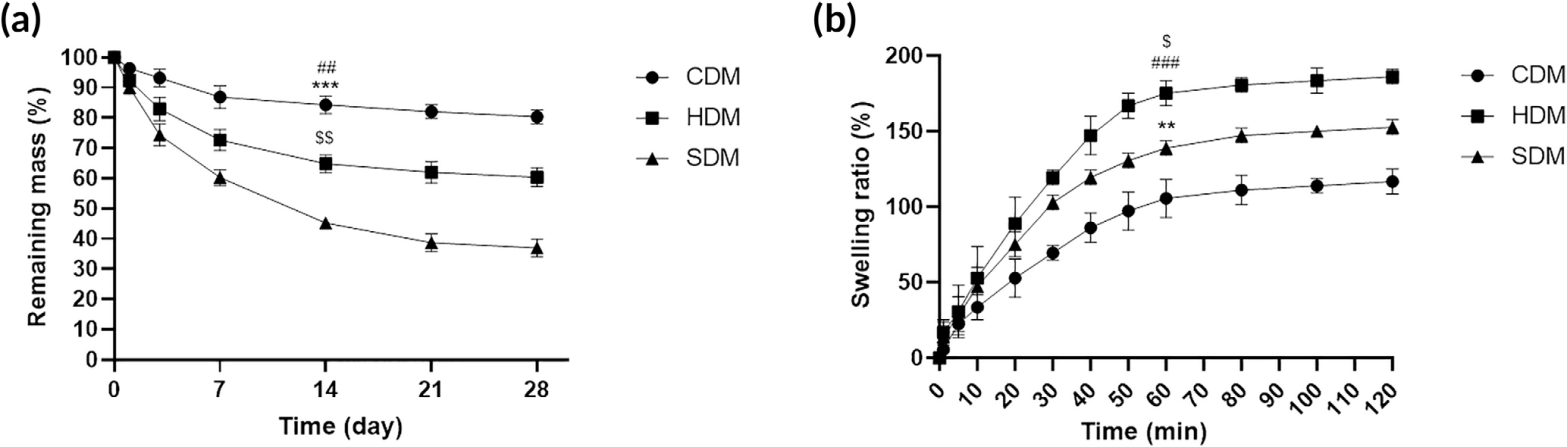
In vitro degradation and hydration behavior of decellularized meniscal scaffolds over time. (a) Enzymatic degradation profile presented as remaining mass (%) over 28 days. Statistical comparisons shown at the representative plateau time point (day 14); intergroup differences remained consistent thereafter. Statistical significance: ## *p*< 0.01 (CDM vs. HDM), ***p< 0.001 (CDM vs. SDM), $$ *p*< 0.01 (HDM vs. SDM). (b) Swelling behavior presented as swelling ratio (%) over 120 min. Statistical comparisons shown at the representative plateau time point (60 min); swelling profiles remained relatively stable thereafter. Statistical significance: $ *p*< 0.05 (HDM vs. SDM), ### *p*< 0.001 (HDM vs. CDM) and **p< 0.01 (SDM vs. CDM). Mean ± SD with individual data points (*n* = 3/group); two‐way ANOVA with Tukey's post‐hoc test. CDM, camel decellularized meniscus; HDM, human decellularized meniscus; SDM, sheep decellularized meniscus.

All decellularized scaffolds exhibited gradual mass loss during enzymatic degradation, with CDM retaining significantly greater remaining mass at the representative plateau (day 14: 84.31% ± 2.97%) compared to HDM (64.88% ± 2.93%) and SDM (45.23% ± 1.96%; Figure 6a). Conversely, HDM scaffolds demonstrated the highest equilibrium swelling ratio at 60 min (175.00% ± 8.33%), followed by SDM (138.89 ± 4.81%) and CDM (105.55% ± 12.72%; Figure 6b), with all groups reaching hydration equilibrium within 80–120 min.

### 3.7 Cytotoxicity assay

**FIGURE 7:**
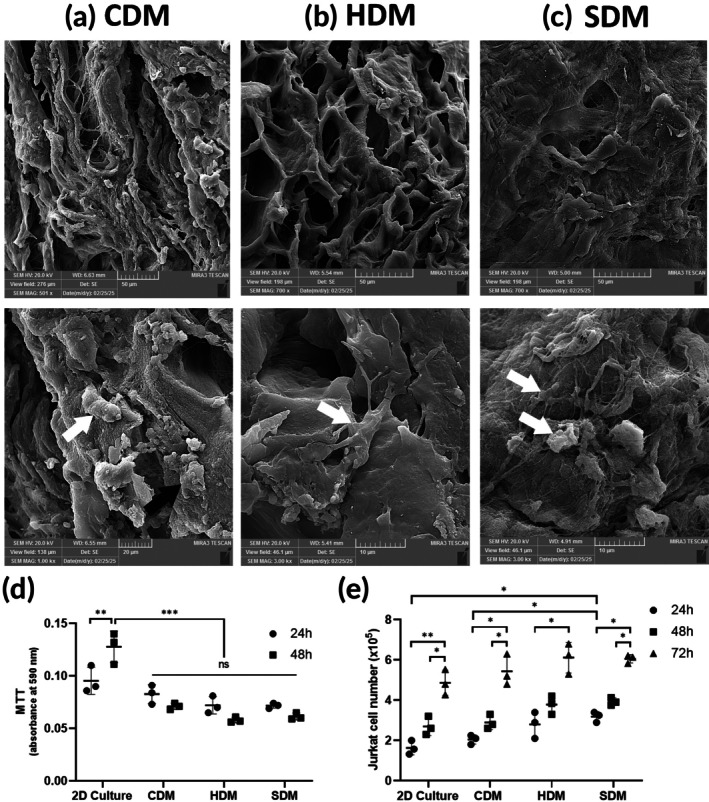
SEM imaging of fibroblast attachment, MTT assay, and Jurkat T‐cell proliferation on decellularized meniscal scaffolds. (a)–(c) SEM images of fibroblasts on CDM, HDM, and SDM scaffolds at high and low magnifications. Arrows indicate attached cells. (d) MTT assay at 24 and 48 h. Two‐dimensional culture showed significantly greater viability at 48 h; no significant differences were observed among 3D scaffold groups or between timepoints, indicating low scaffold cytotoxicity. (e) Jurkat T‐cell proliferation assay at 24, 48, and 72 h on different scaffolds compared with 2D culture controls. Mean ± SD with individual data points (*n* = 3/group); two‐way ANOVA with Šidák's post‐hoc test (*p< 0.05, **p< 0.01, ***p< 0.001; ns, non‐significant).

SEM imaging confirmed fibroblast adhesion and spreading on all decellularized scaffolds after 48 h (Figure 7a–c). MTT assays revealed that cell viability was consistently lower in cells cultured on the scaffolds than in 2D controls at 24 and 48 h. No significant differences were observed among the scaffold groups at either time point. The absence of a notable decrease in viability between the scaffolds at 24 and 48 h indicates that the decellularized scaffolds are nontoxic and generally biocompatible (Figure 7d).

### 3.8 Proliferation of Jurkat cells on the scaffolds

In vitro evaluation revealed that Jurkat cell proliferation in the presence of decellularized scaffolds differed from 2D culture. Cell numbers increased significantly over time (24, 48, and 72 h) under all conditions. Notably, among the scaffold groups, CDM consistently exhibited the lowest mean proliferation across time points under these in vitro conditions, though differences between scaffold groups did not reach statistical significance at 48 and 72 h (Figure 7e).

### 3.9 Raman spectroscopy

**FIGURE 8:**
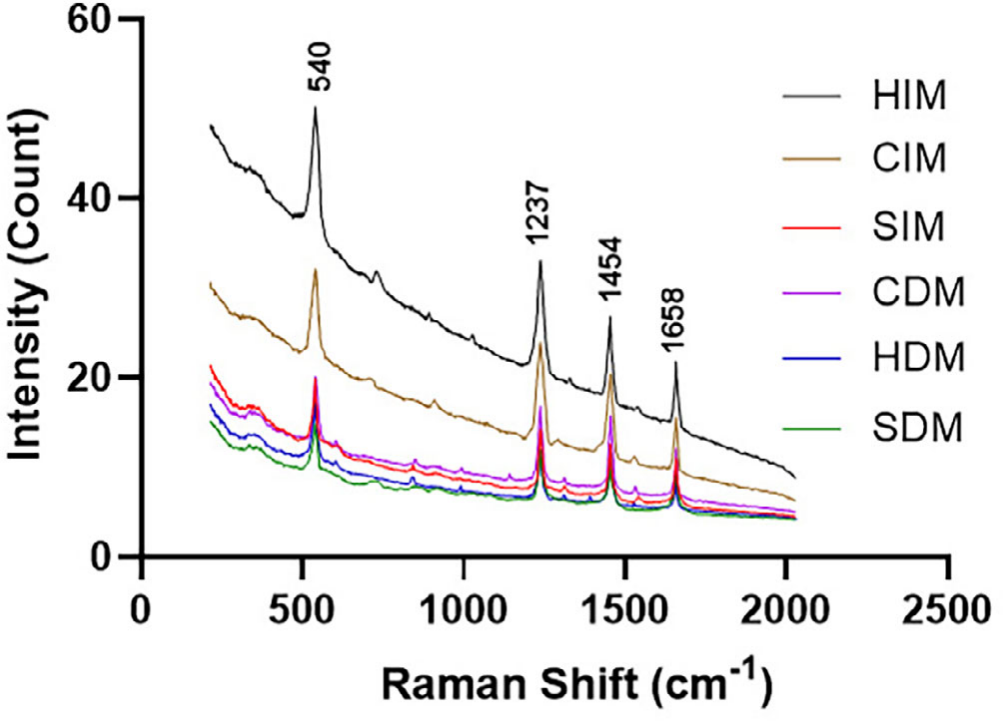
Confocal Raman spectra of intact and decellularized meniscal samples showing Raman shifts (cm^−1^) on the *x*‐axis and intensities (counts) on the *y*‐axis. The prominent peaks at 540, 1237, 1454, and 1658 cm^−1^ correspond to glucose‐saccharide bands, amide III, CH_3_/CH_2_ vibrations, and amide I (collagen‐like proteins), respectively.

Confocal Raman spectroscopy revealed the preservation of the biochemical components of all the decellularized scaffolds in the spectral profiles, which were found to be closely related to those of the intact meniscus. However, the Raman signal intensity was lower in the decellularized scaffolds, which may be due to the removal of the cellular ECM content following decellularization. The spectra exhibited peaks at 540 and 1237 cm^−1^, which were assigned to glucose‐saccharide bands and amide III, respectively. A bond at 1454 cm^−1^ is attributed to overlapping CH_3_ and CH_2_, which are elastin, collagen, and phospholipids. Additionally, a peak at 1658 cm^−1^ was assigned to amide I, corresponding to the presence of collagen‐like proteins^20^ (Figure 8).

## 4 DISCUSSION

Here, we characterized whole decellularized meniscal scaffolds derived from human, camel, and sheep tissues, generating a species‐specific scaffold that preserves native meniscal architecture for use in meniscal tissue engineering. Our findings revealed that the collagen content of decellularized menisci was preserved, with significant removal of GAGs by decellularization. Because GAGs are highly water‐soluble, they are readily lost during decellularization in aqueous solutions. Nevertheless, increased matrix porosity occurred without changing the 3D structure of the scaffold, and may contribute to the reduced mechanical strength of the decellularized scaffold, consistent with findings by Zhang et al.^21^ The reduction in GAG content in the ECM scaffold after decellularization has been widely reported and clearly displays the sensitivity of sulfated proteoglycans to detergent‐based processes.^22^ Despite a decrease in the amount of ECM constituents, the scaffolds maintained sufficient biomimetic capacity to support cell viability and mechanical load bearing. Decellularization has shown special benefits in reducing immunogenicity and conserving the ECM for regeneration of damaged organs.^5^ Subcutaneous implantation of a biocompatible porous decellularized porcine menisci has shown minimal immune response and promoted ECM preservation, along with increased chondrocyte proliferation and synthesis of GAG and type II collagen.^23^ Optimized decellularization and recellularization techniques for meniscus tissue maintain the  ECM without considerable mechanical loss. Acellular meniscal scaffolds prevent immune responses and provide an appropriate microenvironment for the adhesion and proliferation of fibroblasts, chondrocytes, and fibrochondrocytes.^24^

Biocompatibility without a significant immune response was verified in the present study after 4 weeks of subcutaneous implantation of the CDM in this xenogeneic rat model. Enhanced fibroblast migration to CDM may be attributed to the denser collagen fiber packing and superior preservation of native fiber architecture following decellularization, which could provide abundant integrin‐binding sites and contact guidance cues known to facilitate fibroblast infiltration.^25^ While porosity values among decellularized groups showed no significant inter‐species differences, the preserved collagen scaffold likely creates a more permissive microenvironment for cellular integration and tissue remodeling. The combination of reduced inflammatory cell accumulation and increased fibroblast infiltration observed in CDM scaffolds at week 4 aligns with established principles of constructive remodeling in decellularized ECM scaffolds.^26^ Similar patterns of endogenous cell infiltration into acellular meniscal scaffolds have been reported in both PDGF‐enhanced^27^ and growth factor‐free^28^ xenogeneic models, supporting the biological plausibility of our observations. We emphasize that inflammatory cell accumulation was comparable across all three xenogeneic scaffolds at week 1, indicating similar initial immunogenicity. The advantage of CDM therefore lies not in reduced early immune activation, but in its capacity to support timely inflammation resolution and host cell integration—a critical determinant of scaffold remodeling success.

Importantly, all three scaffold types functioned as xenografts in this rodent model; in human recipients, HDM would serve as an allograft with species‐matched major histocompatibility complexes and fundamentally different immune recognition. Consequently, CDM should be viewed as a regionally feasible xenogeneic alternative where human allografts are scarce and porcine sources are inaccessible—not as immunologically superior to human tissue for clinical transplantation. Jurkat T‐cell proliferation assays revealed that all decellularized scaffolds stimulated greater T‐cell expansion than 2D culture during the first 72 h, confirming an initial immune‐activating capacity common to xenogeneic ECM scaffolds. Although CDM exhibited a trend toward lower mean proliferation rates across time points, inter‐group differences did not reach statistical significance—precluding definitive claims of reduced immunogenicity.^24^

Our biomechanical findings align with prior reports that the compressive modulus of decellularized groups was lower than that of corresponding intact tissues, a finding typically attributed to the water‐soluble nature of GAGs and their partial removal during aqueous processing.^16^, ^29^, ^30^ Despite this reduction, the compressive properties of CDM remained comparable to those of HIM, with the ~20% difference not reaching statistical significance. We propose that this preserved mechanical competence is supported by two key compensatory mechanisms: (1) the retention of the circumferential collagen architecture and radial fibers, which provides critical structural confinement against lateral tissue expansion under compression,^31^ thereby maintaining load‐bearing capacity despite increased porosity; and (2) the inherently higher native collagen density of camel meniscus (Figure 2h), which our protocol effectively preserved across all species. Furthermore, the compressive modulus of meniscal tissue is inherently strain‐rate‐dependent due to its biphasic, viscoelastic nature, with dynamic tangent moduli exceeding equilibrium values due to transient fluid pressurization. Chia and Hull^32^ demonstrated ~8‐fold higher moduli at physiological strain rates, and recent studies report dynamic moduli of 2.2–2.5 MPa at 10% strain.^19^ Our reported value of 1.4 MPa for intact human meniscus therefore represents a dynamic tangent modulus consistent with established rate‐dependent behavior, validating the physiological relevance of our testing methodology. No significant differences in UTS were found between the intact and decellularized groups, possibly because the natural orientation of the collagen fibers was preserved following decellularization. While exact compressive modulus matching is often emphasized, clinical success depends more on restoring physiological load distribution than on mechanical equivalence. Meniscal allografts and synthetic implants (e.g., NUsurface®) achieve functional recovery through anatomical contact restoration rather than stiffness replication.^33^, ^34^ Post‐implantation, scaffold mechanics dynamically evolve via host‐driven remodeling. CDM's preserved circumferential collagen architecture guides fibroblast infiltration and oriented neocollagen deposition, gradually restoring tensile competence.^35^ Concurrently, physiological loading promotes GAG accumulation, which can enhance compressive resistance in dynamic conditioning models.^31^ Decellularized collagen fibers were considerably thicker than those in native tissues, likely reflecting reduced interfibrillar tethering following proteoglycan and GAG removal. This loss of matrix constraints allows previously compacted fibrils to relax and swell, a phenomenon consistent with the *“interfibrillar opening”* described in decellularized soft tissues.^36^ Cell‐free ECM scaffolds are designed for gradual remodeling by host cells. Although beneficial tissue regeneration involves some ECM degradation, generally, ECM scaffolds derived from natural materials degrade much more slowly than they do during tissue reconstruction proceeds.^37^ Elsaesser et al. reported that decellularized cartilage is solid in structure, well integrated with native cartilage, and shows minimal infiltration of lymphocytes and macrophages.^38^ Similarly, a slight reduction in CDM was observed 4 weeks after subcutaneous implantation, which was likely related to the infiltration of fibroblasts, ECM biosynthesis, and collagen fiber organization. These findings support Ying‐Chen's hypothesis that the deposition of new ECM by migrating cells may diminish scaffold degradation.^23^ Histomorphometric analysis of remaining scaffold area demonstrated greater structural preservation of CDM scaffolds throughout the implantation period, indicating slower in vivo degradation compared to HDM and SDM scaffolds. Similarly, evaluation of remaining mass during in vitro enzymatic degradation revealed greater structural resistance of CDM scaffolds against matrix breakdown. This slower degradation behavior may be associated with species‐specific collagen crosslinking patterns and the denser collagen fiber packing retained after decellularization, which could reduce enzymatic penetration and delay scaffold degradation. Preservation of scaffold integrity during the early remodeling phase may provide improved mechanical support for progressive tissue regeneration. HDM scaffolds exhibited significantly greater swelling ratios compared to CDM scaffolds. Although SEM analysis did not reveal substantial differences in pore morphology among the groups, hydration behavior is influenced not only by pore structure but also by matrix organization and collagen fiber packing density. Since ECM composition, GAG preservation, and fiber orientation were relatively similar among the scaffolds, the lower swelling ratio observed in CDM scaffolds is more likely associated with their denser collagen architecture. A more compact collagen network may limit water penetration and reduce fluid uptake capacity compared to the less densely packed HDM scaffolds. Importantly, despite the lower swelling ratio, robust fibroblast migration and new collagen deposition within CDM scaffolds at week 4 confirmed that hydration remained functionally sufficient to support cellular viability and tissue integration under physiological conditions. This behavior aligns with studies demonstrating that intact collagen networks provide mechanical confinement against proteoglycan‐driven osmotic swelling, thereby regulating scaffold hydration independently of pore geometry or GAG content.^39^, ^40^ Raman spectral analysis revealed that specific collagen peaks were preserved in decellularized scaffolds. These peaks provide molecular markers of scaffold stability, thus enabling more precise identification and batch verification.

This study has several limitations that should be acknowledged. First, the subcutaneous implantation model cannot replicate the complex biomechanical and immunological environment of the knee joint, limiting assessment of functional scaffold maturation and anisotropic tissue remodeling under physiological loading. Second, the absence of cyclic fatigue testing under physiologically relevant loading conditions limits our ability to capture dynamic wear, macrophage‐mediated remodeling, or long‐term mechanical durability. While static characterization establishes baseline mechanical and degradation competence, dynamic fatigue assessment is widely recognized as essential for validating scaffold stability prior to intra‐articular implantation and clinical translation.^41^, ^42^, ^43^ Third, the molecular mechanisms underlying CDM's favorable remodeling profile—particularly macrophage polarization and cytokine signaling—were not characterized. Finally, quantitative thresholds for acceptable post‐implantation compressive modulus mismatch remain undefined in the literature. Because subcutaneous implantation cannot replicate complex intra‐articular loading, longitudinal studies in load‐bearing environments are essential to define clinically acceptable modulus mismatch thresholds and validate long‐term mechanical maturation.

## 5 CONCLUSION

For the first time, this comparative study evaluated whole decellularized meniscal scaffolds from camels, humans, and sheep. The optimized protocol successfully eliminated cellular components while preserving critical ECM architecture, maintaining essential mechanical properties, and demonstrating favorable biocompatibility and non‐toxicity. In this xenogeneic rat model, the CDM scaffold exhibited favorable remodeling characteristics including reduced inflammatory infiltration, fibroblast migration into the scaffold interior, and slower degradation after 4 weeks compared to the more rapid breakdown observed in human and sheep scaffolds. Notably, camel meniscus morphology has not been previously characterized in the scientific literature; our study establishes the first quantitative anatomical dataset for Camelus dromedarius menisci and demonstrates that CDM scaffolds closely approximate human dimensions while maintaining compressive properties comparable to native tissue. Given the regional constraints discussed earlier, CDM represents a promising xenogeneic scaffold source worthy of further translational investigation. Human allografts remain the clinical gold standard when available; CDM should be viewed as a pragmatic alternative for settings where conventional sources are inaccessible. Advancing CDM toward clinical application will necessitate orthotopic implantation in large‐animal models, where dynamic loading protocols must be employed to verify structural resilience under functional joint stresses.

## AUTHOR CONTRIBUTIONS

**Mohammad Mehdi Hassanzadeh‐Taheri:** Writing – original draft. **Hamid Hashemi:** Methodology. **Mohammad Afshar:** Supervision; project administration. **Saeed Vafaei‐Nezhad:** Methodology. **Mohsen Rezaeipour:** Methodology. **Mohammad Reza Khakzad:** Project administration. **Tahereh Talaei‐Khozani:** Writing – review and editing; data curation. **Mehri Shadi:** Funding acquisition; writing – original draft; investigation.

## FUNDING INFORMATION

This study was financially supported by the Research Deputy of Birjand University of Medical Sciences and the Innovation Research Center, Islamic Azad University, Mashhad University of Medical Sciences (Grant No. 2899).

## CONFLICT OF INTEREST STATEMENT

The authors declare that there are no conflicts of interest regarding the publication of this paper.

## Data Availability

The data that support the findings of this study are available from the corresponding author upon reasonable request.
